# Association with HLA-DRβ1 position 37 distinguishes juvenile dermatomyositis from adult-onset myositis

**DOI:** 10.1093/hmg/ddac019

**Published:** 2022-01-31

**Authors:** Claire T Deakin, John Bowes, Lisa G Rider, Frederick W Miller, Lauren M Pachman, Helga Sanner, Kelly Rouster-Stevens, Gulnara Mamyrova, Rodolfo Curiel, Brian M Feldman, Adam M Huber, Ann M Reed, Heinrike Schmeling, Charlotte G Cook, Lucy R Marshall, Meredyth G Ll Wilkinson, Stephen Eyre, Soumya Raychaudhuri, Lucy R Wedderburn, the Juvenile Dermatomyositis Cohort and Biomarker Study, the Childhood Myositis Heterogeneity Study Group, and the Myositis Genetics Consortium (MYOGEN)

**Affiliations:** Infection, Immunity and Inflammation Research and Teaching Department, UCL Great Ormond Street Institute of Child Health, London, UK; Centre for Adolescent Rheumatology Versus Arthritis at UCL, UCL Hospital and Great Ormond Street Hospital, London, UK; NIHR Biomedical Research Centre at Great Ormond Street Hospital, London, UK; Centre for Genetics and Genomics Versus Arthritis, Centre for Musculoskeletal Research, Manchester Academic Health Science Centre, The University of Manchester, Manchester, UK; National Institute of Health Research Manchester Biomedical Research Centre, Manchester Academic Health Science Centre, Manchester University NHS Foundation Trust, Manchester, UK; Environmental Autoimmunity Group, Clinical Research Branch, National Institute of Environmental Health Sciences, National Institutes of Health, Bethesda, MD, USA; Environmental Autoimmunity Group, Clinical Research Branch, National Institute of Environmental Health Sciences, National Institutes of Health, Bethesda, MD, USA; Ann & Robert H. Lurie Children’s Hospital of Chicago, Feinberg School of Medicine, Northwestern University, Chicago, IL, USA; Department of Rheumatology, University of Oslo, Oslo, Norway; Oslo New University College, Oslo, Norway; Emory University School of Medicine, Atlanta, GA, USA; Division of Rheumatology, George Washington University School of Medicine and Health Sciences, Washington, DC, USA; Division of Rheumatology, George Washington University School of Medicine and Health Sciences, Washington, DC, USA; Division of Rheumatology, Department of Pediatrics, The Hospital for Sick Children, Toronto, ON, Canada; IWK Health Centre and Dalhousie University, Halifax, NS, Canada; Pediatrics, Duke University, Durham, NC, USA; Department of Pediatrics, Alberta Children’s Hospital, University of Calgary, Calgary, AB, Canada; Infection, Immunity and Inflammation Research and Teaching Department, UCL Great Ormond Street Institute of Child Health, London, UK; Infection, Immunity and Inflammation Research and Teaching Department, UCL Great Ormond Street Institute of Child Health, London, UK; Centre for Adolescent Rheumatology Versus Arthritis at UCL, UCL Hospital and Great Ormond Street Hospital, London, UK; NIHR Biomedical Research Centre at Great Ormond Street Hospital, London, UK; Infection, Immunity and Inflammation Research and Teaching Department, UCL Great Ormond Street Institute of Child Health, London, UK; Centre for Adolescent Rheumatology Versus Arthritis at UCL, UCL Hospital and Great Ormond Street Hospital, London, UK; NIHR Biomedical Research Centre at Great Ormond Street Hospital, London, UK; Centre for Genetics and Genomics Versus Arthritis, Centre for Musculoskeletal Research, Manchester Academic Health Science Centre, The University of Manchester, Manchester, UK; National Institute of Health Research Manchester Biomedical Research Centre, Manchester Academic Health Science Centre, Manchester University NHS Foundation Trust, Manchester, UK; Centre for Genetics and Genomics Versus Arthritis, Centre for Musculoskeletal Research, Manchester Academic Health Science Centre, The University of Manchester, Manchester, UK; National Institute of Health Research Manchester Biomedical Research Centre, Manchester Academic Health Science Centre, Manchester University NHS Foundation Trust, Manchester, UK; Department of Medicine, Brigham and Women's Hospital and Harvard Medical School, Boston, MA, USA; Program in Medical and Population Genetics, Broad Institute of MIT and Harvard, Cambridge, MA, USA; Infection, Immunity and Inflammation Research and Teaching Department, UCL Great Ormond Street Institute of Child Health, London, UK; Centre for Adolescent Rheumatology Versus Arthritis at UCL, UCL Hospital and Great Ormond Street Hospital, London, UK; NIHR Biomedical Research Centre at Great Ormond Street Hospital, London, UK

## Abstract

Juvenile dermatomyositis (JDM) is a rare, severe autoimmune disease and the most common idiopathic inflammatory myopathy of children. JDM and adult-onset dermatomyositis (DM) have similar clinical, biological and serological features, although these features differ in prevalence between childhood-onset and adult-onset disease, suggesting that age of disease onset may influence pathogenesis. Therefore, a JDM-focused genetic analysis was performed using the largest collection of JDM samples to date. Caucasian JDM samples (*n* = 952) obtained via international collaboration were genotyped using the Illumina HumanCoreExome chip. Additional non-assayed human leukocyte antigen (HLA) loci and genome-wide single-nucleotide polymorphisms (SNPs) were imputed. HLA-DRB1^*^03:01 was confirmed as the classical HLA allele most strongly associated with JDM [odds ratio (OR) 1.66; 95% confidence interval (CI) 1.46, 1.89; *P* = 1.4 × 10^−14^], with an independent association at HLA-C^*^02:02 (OR = 1.74; 95% CI 1.42, 2.13, *P* = 7.13 × 10^−8^). Analyses of amino acid positions within *HLA-DRB1* indicated that the strongest association was at position 37 (omnibus *P* = 3.3 × 10^−19^), with suggestive evidence this association was independent of position 74 (omnibus *P* = 5.1 × 10^−5^), the position most strongly associated with adult-onset DM. Conditional analyses also suggested that the association at position 37 of *HLA-DRB1* was independent of some alleles of the Caucasian HLA 8.1 ancestral haplotype (AH8.1) such as HLA-DQB1^*^02:01 (OR = 1.62; 95% CI 1.36, 1.93; *P* = 8.70 × 10^−8^), but not HLA-DRB1^*^03:01 (OR = 1.49; 95% CR 1.24, 1.80; *P* = 2.24 × 10^−5^). No associations outside the HLA region were identified. Our findings confirm previous associations with AH8.1 and HLA-DRB1^*^03:01, HLA-C^*^02:02 and identify a novel association with amino acid position 37 within *HLA-DRB1*, which may distinguish JDM from adult DM.

## Introduction

Juvenile dermatomyositis (JDM) is a rare, severe autoimmune disease and the most prevalent idiopathic inflammatory myopathy with proximal muscle weakness and skin rash as typical features. Clinical features are heterogeneous and can include serious complications such as calcinosis, ulceration, treatment-resistant rash and involvement of major organs, including gut, lungs and brain. Although some patients achieve remission following standard disease management, which consists of long-term immunosuppression with glucocorticoids, methotrexate and other medications, others respond poorly.

While JDM and adult-onset dermatomyositis (DM) share similar clinical and biological features, there are differences in prevalence of clinical features (1). The incidence of JDM is approximately one-tenth of the incidence of DM (2). DM can be associated with cancer, but this has not been reported in JDM. Conversely, calcinosis is a major cause of morbidity in JDM but has a lower prevalence in DM. The prevalence of myositis-specific autoantibodies (MSAs), which are linked to different clinical features of disease, also differs between the adult and juvenile forms of the disease. Anti-nuclear matrix protein-2 is one of the more abundant MSAs in JDM (reported in 20–25% of patients (3–5)) but has a lower prevalence in DM (reported in 1.6–17% of different patient populations) (6–8). The most prevalent MSA in DM, anti-histidyl tRNA synthetase (anti-Jo-1), is rare in JDM. These differences in the distribution of MSA and clinical features suggest an influential role for age of disease onset in the pathogenesis of disease. However, little is known at the mechanistic level about the influence of age on JDM phenotypes and pathogenesis. Knowledge about how disease mechanisms differ between patient subgroups and interact with patient age to result in different complications may enable targeting of novel molecular pathways, more accurate modelling of life-long risk and more stratified therapeutic approaches to address this risk.

Candidate gene and genome-wide studies of myositis have established the strongest genetic association within the Caucasian 8.1 ancestral haplotype (AH8.1; HLA A1-B8-DR3-DQ2) of the major histocompatibility complex (MHC), also associated with many other immune-mediated diseases (9–13). Distinct human leukocyte antigen (HLA) alleles have been identified as associated with serological subphenotypes of myositis in different ethnic populations. Most notably associations between the development of anti-Jo-1 autoantibodies and HLA-DRB1^*^03:01, HLA-DQB1^*^02:01 and HLA-B^*^08, consistent with AH8.1, have been identified in Caucasian and African-American patients (11,12,14). In adult myositis, gene–environment interactions have been found between HLA-DRB1^*^03, smoking and the presence of anti-Jo-1 autoantibodies, and between HLA-DRB1^*^11:01 and anti-3-hydroxy-3-methylglutaryl-CoA reductase (anti-HMGCR)-positive statin-induced immune-mediated necrotising myopathy (15,16). To date, the rarity of JDM has meant that candidate gene studies in JDM have been small and subgroup analyses of JDM in genome-wide studies have had limited statistical power relative to other myositis phenotypes. The aim of this research was to identify novel genetic loci associated with JDM using a larger cohort of patients.

## Results

### Samples and genotyping quality control

Samples of Caucasian ancestry (*n* = 952) were obtained via international collaboration including samples from the UK Juvenile Dermatomyositis Cohort & Biomarker Study, the Childhood Myositis Heterogeneity Study Group and the Myositis Genetics Consortium (Table 1). Many of these cases have contributed to previous analyses (10,17). Demographic features are described in Supplementary Material, Table S1. After genotyping and stringent quality control (QC), *n* = 178 164 single-nucleotide polymorphisms (SNPs) remained (Supplementary Material, Table S2) on *n* = 851 JDM samples (Supplementary Material, Tables S3 and S4). The proportion of phenotypic variance explained by these markers was estimated as 0.18 (0.02).

**Table 1:** Sources of samples from Caucasian patients with JDM

Country	Source	Genotyped samples (*n* = 952)	Genotyped samples after quality control (*n* = 851)
UK	UK Juvenile Dermatomyositis Cohort & Biomarker Study	365	326
USA	National Institute of Environmental Health Sciences	262	224
	Northwestern University	140	116
	Emory University	41	31
	George Washington University	32	30
	Mayo Clinic	13	12
Canada	The Hospital for Sick Children, Toronto	24	24
	IWK Health Centre	16	16
	Alberta University	10	9
Norway	Oslo University Hospital	49	46

### HLA-DRB1^*^03:01 confirmed as allele most strongly associated with JDM

**Figure 1:**
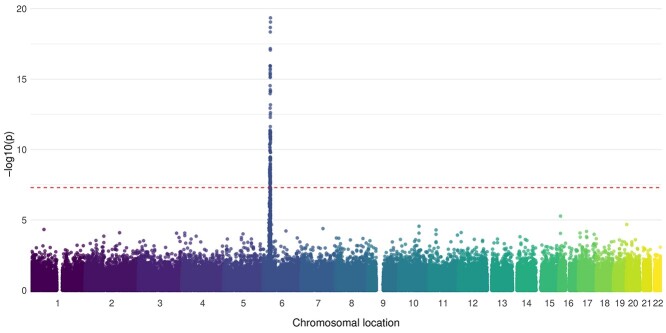
Manhattan plot of the association of assayed SNPs with JDM. SNPs (*n* = 178 164) were available for *n* = 851 JDM samples and *n* = 12 232 controls of Caucasian origin. Association was tested using logistic regression, with the first 10 principal components included as covariates to account for population stratification. The red dotted line indicates genome-wide level of significance (5 × 10^−8^).

Case–control analysis of assayed SNPs confirmed that the strongest association with JDM was within the HLA region (Fig. 1; Supplementary Material, Fig. S1). Analysis of imputed markers within the HLA region indicated that the strongest association was with SNP rs3117103 [odds ratio (OR) = 1.87; 95% confidence interval (CI) 1.64, 2.13; *P* = 1.79 × 10^−20^] and the classical allele HLA-DRB1^*^03:01, consistent with previous reports (9,10). The OR for HLA-DRB1^*^03:01 itself was 1.66 (95% CI 1.46, 1.89; *P* = 1.4 × 10^−14^; Supplementary Material, Table S5).

Conditioning on this allele, an independent association with HLA-C^*^02:02 was identified (OR = 1.74; 95% CI 1.42, 2.13, *P* = 7.13 × 10^−8^) as reported previously (10). The next most associated allele was HLA-B^*^44:02, but the *P*-value for this association was above the threshold for statistical significance (OR = 1.31, 95% CI 1.15, 1.50; *P* = 6.04 × 10^−5^). No alleles within *HLA-A* were associated with JDM (all *P*-values above 0.08).

### Analysis of amino acid positions within *HLA-DRB1* identifies position 37 as the most significantly associated with JDM

**Figure 2:**
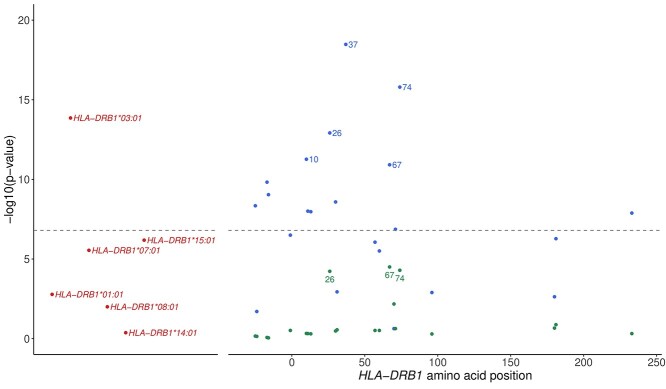
Representation of the *P*-values for omnibus tests of individual amino acid positions within *HLA-DRB1* for association with JDM. Blue circles represent *P*-values for amino acid positions tested alone. Green circles represent *P*-values for amino acid positions after conditioning on position 37. Red circles represent *P*-values for 4-digit classical alleles. The grey dashed line represents the threshold for statistical significance (*P* = 6.8 × 10^−6^).

Since the strongest association with JDM was identified within *HLA-DRB1*, further analysis sought to resolve this association at the level of amino acid positions. Position 37 was the most strongly associated with disease (omnibus *P* = 3.3 × 10^−19^; Fig. 2 and Table 2). Residues at position 37 are located within the P9 pocket of the antigen-binding groove (Supplementary Material, Fig. S2) (18,19). Relative to Tyr-37, Ser-37 was the most significantly associated residue at this position (OR = 0.65; 95% CI 0.57, 0.75; *P* = 7.34 × 10^−10^), followed by Phe-37 (OR = 0.63; 95% CI 0.54, 0.75; *P* = 1.14 × 10^−7^) (Table 3). Asn-37, which is found on HLA-DRB1^*^03:01, was not associated relative to Tyr-37 (OR = 1.13; 95% CI 1.06, 1.19; *P* = 0.05). Conditioning on position 37, there was suggestive evidence of possible independent associations at positions 74 (*P* = 5.1 × 10^−5^) and 26 (*P* = 5.9 × 10^−5^), although the *P*-values for these associations were not significant (Table 2).

**Table 2:** Analysis of amino acid positions within *HLA-DRB1* associated with JDM^a^

			Effects independent of position 37	
Position	χ^2 b^	*P*-value	χ^2^	*P*-value
37	89.2	3.3 × 10^−19^	–	–
74	76.7	1.6 × 10^−16^	22.5	5.1 × 10^−05^
26	59.5	1.2 × 10^−13^	19.5	5.9 × 10^−05^
10	47.5	5.4 × 10^−12^		
67	46.0	1.2 × 10^−11^		
−17	41.0	1.5 × 10^−10^		
−16	37.5	9.1 × 10^−10^		
30	39.5	2.6 × 10^−9^		
−25	34.4	4.5 × 10^−9^		
11	40.2	9.9 × 10^−9^		
13	40.0	1.1 × 10^−08^		
233	32.3	1.3 × 10^−8^		
71	27.8	1.4 × 10^−7^		
−1	26.1	3.2 × 10^−7^		
181	28.9	5.3 × 10^−7^		
57	24.2	8.8 × 10^−7^		
60	25.4	3.1 × 10^−6^		

^a^Alpha threshold for statistical significance in the MHC region taken as 6.8 × 10^−6^ (47).

^b^Test statistics compared using a chi-squared distribution.

**Table 3:** Association of residues within position 37 with JDM, with effects of classical HLA alleles linked to these residues^a,^^b^

Residue	Classical HLA allele linked to residue	Allele frequency in JDM	Allele frequency in controls	OR	95% CI	*P*-value
Tyr-37	–	0.37	0.31	-	-	-
Ser-37	–	0.21	0.26	0.65	0.57, 0.75	7.3 × 10^−10^
–	HLA-DRB1^*^01:01	0.10	0.12	0.76	0.65, 1.46	1.7 × 10^−3^
–	HLA-DRB1^*^15:01	0.11	0.14	0.66	0.57, 0.78	6.5 × 10^−7^
Phe-37	–	0.11	0.15	0.63	0.54, 0.75	1.1 × 10^−7^
–	HLA-DRB1^*^07:01	0.09	0.13	0.67	0.57, 0.79	2.8 × 10^−6^
–	HLA-DRB1^*^14:01	0.02	0.02	0.86	0.60, 1.24	0.42
Asn-37	–	0.31	0.24	1.13	1.06, 1.19	0.05
–	HLA-DRB1^*^03:01	0.19	0.12	1.66	1.46, 1.89	1.4 × 10^−14^

^a^Summary statistics for individual residues derived from the omnibus model for position 37, with Tyr-37 used as the reference residue. Summary statistics for classical four-digit HLA alleles derived from case–control analysis of imputed HLA data. Alleles linked to these residues at position 37 but not represented were filtered out during QC.

^b^Alpha threshold for statistical significance in the MHC region taken as 6.8 × 10^−6^ (47).

To evaluate whether position 37 explained the association within *HLA-DRB1* more convincingly than the classical HLA-DRB1^*^03:01 allele and AH8.1, conditional analyses were performed. Conditioning on HLA-DRB1^*^03:01, the association of position 37 was above the threshold for significance (omnibus *P* = 4.42 × 10^−5^), although there may be weak evidence of an independent association. After conditioning on all residues at position 37, there was evidence of independent effects for HLA-DQB1^*^02:01 within AH8.1 (OR = 1.62; 95% CI 1.36, 1.93; *P* = 8.70 × 10^−8^) and HLA-C^*^02:02 (OR = 1.72; 95% CI 1.40, 2.10; *P* = 1.58 × 10^−7^). However, the effect for HLA-DRB1^*^03:01 did not meet the threshold for significance (OR = 1.49; 95% CR 1.24, 1.80; *P* = 2.24 × 10^−5^). Taken together, these results indicate an effect of position 37 that is independent of some of the previously reported effects within AH8.1 and at HLA-C^*^02:02, but that is not independent of HLA-DRB1^*^03:01.

### Genome-wide imputation identifies possible loci associated at a suggestive level of significance

**Figure 3:**
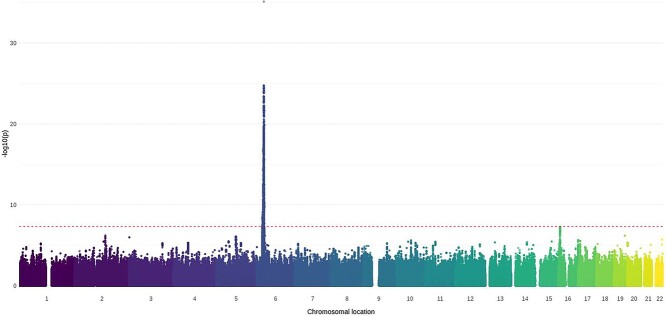
Manhattan plot of the association of imputed SNPs with JDM. Loci were filtered according to MAF of 0.05. Data were imputed for *n* = 851 JDM samples and *n* = 12 232 controls of Caucasian origin. The first 10 principal components were adjusted for during analysis to account for population stratification. SNPs were weighted by the information score to account for imputation uncertainty. The red dotted line indicates genome-wide level of significance (5 × 10^−8^). The degree of transparency of each data-point represents the *R*^2^ value for imputation accuracy, with more solid colours representing higher certainty.

Following genome-wide imputation, there were no additional loci identified at the genome-wide level of statistical significance (*P* = 5 × 10^−8^). Two loci with *P* < 1 × 10^−6^ and minor allele frequency (MAF) over 0.05 were identified (Table 4; Fig. 3). rs6501160 is an intronic variant within *transmembrane protein 114* (*TMEM114*), a transmembrane protein. rs6892006 is an intronic variant within *myocyte enhancer factor 2C antisense RNA 1* (*MEF2C-AS1*), an anti-sense RNA gene (Supplementary Material, Fig. S3). Associations with clinical phenotypes have not been reported for either variant. Although additional loci were identified at *P* < 5 × 10^−8^ (Supplementary Material, Table S6 and Supplementary Material, Fig. S4), these had MAF between 0.01 and 0.05 and may represent imputation artefacts.

**Table 4:** Genome-wide imputed loci displaying potential association with JDM, with *P*-value cut-offs of 1 × 10^−6^ and MAF cut-off of 0.01^a^

Rsid^b^	Chromosome	Position	Allele A	Allele B	MAF	OR	95% CI	*P*-value	Imputation *R*^2 c^	Information measure^d^	Association information measure^e^
rs6501160	16	8 607 139	G	A	0.27	0.72	0.65, 0.81	5.8 × 10^−8^	0.91	0.91	0.91
rs6892006	5	88 450 541	T	G	0.13	0.68	0.58, 0.79	8.7 × 10^−7^	0.87	0.87	0.89

^a^Loci with *P*-values below a suggestive level of significance (1 × 106) are displayed for MAF cut-offs of 0.05 and 0.01; only additional loci captured by the lower MAF cut-off are shown for that cut-off.

^b^Rsid, Reference SNP cluster ID. There were no assayed markers within 100 000 kb of either of these loci.

^c^Squared correlation of imputation of genotypes with true unmeasured genotypes, as estimated by Minimac3 during imputation via the Michigan Imputation Server: https://genome.sph.umich.edu/wiki/Minimac3_Info_File#Rsq.

^d^Impute INFO measure calculated by SNPTEST: https://mathgen.stats.ox.ac.uk/genetics_software/snptest/snptest.html#info_measures.

^e^Relative information measure about parameters of the model fitted during association testing: https://mathgen.stats.ox.ac.uk/genetics_software/snptest/snptest.v2.p.

## Discussion

This analysis represents the largest international genetic study of JDM to date. We found that the strongest association was in the HLA region within HLA-DRB1^*^03:01, consistent with previous studies (9,10,17). We confirmed an independent association with HLA-C^*^02:02 and found some evidence of a possible additional independent association at HLA-B^*^44:02, which would need to be confirmed in a future study. Interestingly, we did not find evidence of an association with *HLA-A*, even though associations within this gene are well known for multiple other autoimmune diseases including rheumatoid arthritis (RA), juvenile idiopathic arthritis (JIA), psoriatic arthritis and type I diabetes (20–23).

In this analysis focused on juvenile-onset disease, we found amino acid position 37 within *HLA-DRB1* had the strongest association with JDM, with Ser-37 and Phe-37 having protective effects relative to Tyr-37. We also found evidence that the association with position 37 was independent of AH8.1, a well-established association with myositis, but there was only weak evidence of independence from HLA-DRB1^*^03:01, and so this needs to be followed up in future studies with greater numbers of both juvenile-onset and adult-onset patients using the same genotyping chip. Amino acid position 37 is within the P9 pocket of the antigen-binding groove (18,19). Substitution of Ser-37 to Tyr-37 has been shown to be sufficient to alter alloantigenicity and stimulate a T-cell response (24). Tyr-37 promotes a stronger response to streptococcal protein (25).

The previous Immunochip analysis of adult and juvenile myositis combined identified the strongest association within *HLA-DRB1* at amino acid position 74 (10). Subsequent analyses of that dataset identified position 74 as having an association with anti-Jo1, anti-PM/Scl and Anti-cytosolic 5′-nucleotidase 1A (anti-cN1A) autoantibodies (26), which are less prevalent in juvenile-onset disease. We found weak evidence of independent association at position 74 after conditioning on position 37, above the threshold for significance and so this finding needs to be followed up with greater number of patients. Nonetheless, it raises an intriguing possibility that children and adults may have different dominant autoantigenic peptides being presented in the antigen-binding groove, although we recognize that multiple antigens with different allelic associations are likely to be involved in disease. While the Immunochip analyses benefitted from a relatively large cohort (*n* = 2544), the number of JDM patients was approximately half that of our analysis (*n* = 493). These patients are represented in our analysis, along with additional cases. It may be that the effects of juvenile-onset disease were obscured by larger number of adult-onset patients and other phenotypes present in the combined cohort. Future work and greater numbers are required to better dissect the genetic influence on age effects in juvenile and adult-onset disease, as well as on clinical and serological heterogeneity.

Associations between other positions within *HLA-DRB1* and autoimmune disease have been reported. For example, position 11 has the strongest effect in RA, with independent effects at positions 71 and 74 (27). In JIA, position 13 has the strongest effect, although in systemic JIA the strongest effect is at position 58 (21,28). Positions 71/74 have also been implicated in anti-fibrillarin-positive systemic sclerosis, Crohn’s disease, multiple sclerosis, type I diabetes and Grave’s disease (29–33). In systemic lupus erythematosus (SLE), Ser-1, Phe47 and Ala71 are associated with disease (34). Position 37 has been found to be associated with primary sclerosing cholangitis, ulcerative colitis in Asians, ACPA^+^ RA in Han Chinese, SLE in Asians and psoriasis vulgaris in Taiwanese (18,19,35–37). Interestingly, a recent report of Japanese patients with RA identified an association between position 37 and younger age of disease onset (defined as 16–30 years of age), but not with older age of onset (defined as over 60 years of age) (38). At present, it is not well understood how these differing positional effects relate to the spectrum of phenotypes represented in autoimmune disease, and how these effects interact with ethnicity and age of disease onset.

The major limitation of our study is limited statistical power for identifying associations outside the HLA region, which is a practical challenge for rare disease research. Although our combined cohort represents the largest-ever assembled international cohort of Caucasian patients with JDM, it is a relatively small sample size for a genetic study. As such, the findings of suggestive associations will need to be confirmed in future studies with greater number of patients. Nonetheless, we were able to confirm the findings of previous studies in a larger cohort dedicated to JDM. Future studies with greater numbers may better define the relationship between the associations at HLA-DRB1^*^03:01 and amino acid positions within *HLA-DRB1* such as position 37. Loci outside the HLA region identified at genome-wide and suggestive levels of significance in previous analyses (including *PTPN22*, *UBE2L3*, *CD28, TRAF6, STAT4*) were not replicated in this study, although signals at these loci were not specifically tested for. This may reflect the smaller sample size of this study and the dominance of adult patients in those analyses whose characteristics may differ from paediatric disease (10). JDM is a heterogeneous phenotype; however, the small sample size restricted our ability to analyse more clinically homogeneous subgroups, such as autoantibody subgroups. It may be that future developments in methodologies for genetic analysis will enable further insights to be derived from this dataset. Many of the patients in this study overlapped with a previous analysis, which identified associations with major MSA subtypes, including anti-Jo-1, anti-PM/Scl, anti-cN1A, anti-Mi-2 and anti-TIF1γ (26). As more patients become available for inclusion in genetic studies, analyses of further MSA subtypes may become possible. It will also be critical to study genetic associations with MSA subtypes in different ethnic populations.

Analysis following genome-wide imputation identified 2 possible suggestive loci, an intronic variant within *TMEM114* and an intronic variant within *MEF2C-AS1*. *TMEM114* is a glycosylated transmembrane protein, and knowledge of its cellular function is limited, although missense mutations in this gene and a chromosomal translocation in its promoter are associated with congenital and juvenile cataract disorders, respectively (39). *MEF2C-AS1* is a non-coding anti-sense RNA gene with no known function. It is unclear how these loci relate to JDM, but it may be these alleles function as epigenetic marks.

In summary, we have confirmed the association between JDM and HLA-DRB1^*^03:01 and shown that within *HLA-DRB1*, position 37 is most strongly associated with disease in a population of patients with juvenile-onset myositis.

## Materials and Methods

### Genotyping and genotype calling

Genotyping of Caucasian JDM samples (*n* = 952) was performed using the Illumina (Cambridge, UK) HumanCoreExome chip across three batches at a single centre (University College London). This cohort included the majority of JDM cases included in previous analyses, as well as additional cases recruited subsequent to those analyses including cases from more centres (10,17). GenomeStudio 2.0 (version 2.0.4 of the Genotyping Module and the GenTrain 3.0 Cluster Algorithm) was used for genotype clustering and calling for each separate batch. Samples with less than 90% call rate were excluded, and genotype clustering and calling were repeated, before data were exported in PLINK format for QC.

Data on healthy individuals (*n* = 12 474) of European ancestry who had also been genotyped using the HumanCoreExome chip as part of the International Age-Related Macular Degeneration Genetic Consortium were obtained from dbGaP (dbGAP Study Accession phs001039.v1.p1).

### Quality control

QC of markers and samples was performed as described previously (22). The following steps were done separately for each of the three batches of JDM data and the control data using PLINK 1.07 (40). Mitochondrial and Y chromosome SNPs were excluded. SNPs were also excluded if they had elevated missing rates (over 2%), had a MAF below 1% and deviated significantly from Hardy–Weinberg equilibrium (HWE; threshold of *P* < 0.0001). Samples with elevated missing genotypes (over 5%) and outlying heterozygosity rates (above or below 5 standard deviations from the mean rate). Data were aligned to the Haplotype Reference Consortium (HRC) before merging for further QC using the HRC checking tool (41).

Duplicated or related individuals in the merged dataset were identified using identity-by-descent, performed on a linkage disequilibrium (LD)-pruned dataset of 65 862 SNPs with MAF over 5%. A PI_HAT threshold of 0.2 was used, with the individual with the most missing data excluded. Principal component analysis was performed using PLINK 1.9 (42), to evaluate population stratification in the LD-pruned data merged with the International HapMap 3 data (43). Cases and controls were retained if they were within 10 standard deviations of the mean value for the first two principal components (PCs) for the HapMap CEU population (Supplementary Material, Fig. S5).

Following QC, there were 178 164 SNPs for *n* = 851 JDM cases and *n* = 12 232 controls (Supplementary Material, Tables S1–S4).

### Analysis of assayed markers

Autosomal markers were analysed for association with JDM using logistic regression in PLINK with adjustment for the first 10 PCs to control for population stratification. Manhattan plots were generated using R version 6.3 and the ‘ggplot2’ package (version 3.3.2). The proportion of phenotypic variance explained by the SNPs and a standard error for that estimated proportion were estimated using the genome-based restricted maximum likelihood (GREML) method using GTAC version 1.93.3, assuming an approximate prevalence of 0.00004 for JDM (2,44,45).

### Imputation of HLA loci

Classical HLA alleles, amino acids and SNPs within the HLA region were imputed using SNP2HLA (version 1.0.3) and the Type 1 Diabetes Genetics Consortium reference panel (*n* = 5225) (46). QC of imputed markers used the following criteria: imputation information score (*R*^2^) over 0.9, MAF over 0.01 and significant departures from HWE in controls (*P* < 0.001). Imputed markers were coded as present or absent.

### Analysis of HLA and amino acid positions

Case–control analysis of all imputed HLA was performed assuming an additive model using logistic regression in PLINK, also with adjustment for the first 10 PCs, to identify the most strongly associated locus. Clinical covariates were not adjusted for. For HLA analyses, *P*-values below 6.8 × 10^−6^ were considered statistically significant, using a Bonferroni correction for the number of imputed markers as reported previously (47). The two- and four-digit classical alleles identified by this locus were subsequently conditioned on in further logistic regression analyses to identify any independent associations.

Amino acid positions were interrogated using likelihood ratio tests (LRTs) in R as follows. At each position, logistic regression models were fitted with all residues in the model, except the residue that was most prevalent in the controls and served as the reference. The first 10 PCs were adjusted for in each of these models. To evaluate whether amino acid positions were associated with disease, LRTs were performed to compare the model that was fitted for the residues at each position against a null model, which comprised the first 10 PCs only. The effect of each amino acid position evaluated using a LRT is represented by the LRT *P*-value, but there is no estimated effect size for the amino acid position generated by this test. Allele frequencies, odds ratios and *P*-values for amino acid residues at key positions are also reported. Possible independent effects were identified by conditional analysis as above.

### Genome-wide imputation and analysis

Genome-wide imputation of SNPs was performed using the Michigan Imputation Server (version 1.2.4) and the HRC reference panel (version r1.1; Supplementary Material, Fig. S6) (41,48). Imputed SNPs with MAF below 0.01 or imputation information score below 0.5 were filtered out.

Imputed SNPs were analysed using SNPTEST version 2.5.4-beta3 (49), with adjustment for the first 10 PCs and weighting for the imputation information score to account for imputation uncertainty. Since imputation artefacts are enriched in rare variants, a stringent MAF threshold of 0.05 was used during analysis, although loci with MAF 0.01–0.05 are also reported in Supplementary Material.

## Supplementary Material

Supplementary_information_for_JDM_genetics_manuscript_CD041221_ddac019

HLA_classical_and_amino_acid_summary_statistics_ddac019

## Notes

The authors wish it to be known that, in their opinion, the final two authors should be regarded as joint Senior Authors.
