# Supplementary material for: Association with HLA-DRβ1 position 37 distinguishes juvenile dermatomyositis from adult-onset myositis
Source: Hum Mol Genet. 2022 Jan 31;31(14):2471–81. doi: 10.1093/hmg/ddac019 (PMC9307311; doi:10.1093/hmg/ddac019)
Supplement: Supplementary_information_for_JDM_genetics_manuscript_CD041221_ddac019 [file supplementary_information_for_jdm_genetics_manuscript_cd041221_ddac019.docx]

**Supplementary Material**

**List of tables:**

Supplementary Table S1. Demographic features of n=851 patients contributing samples after quality control

Supplementary Table S2. Numbers of markers following quality control steps

Supplementary Table S3. Numbers of samples following quality control steps performed on separate genotyping batches

Supplementary Table S4. Numbers of samples following quality control steps performed after merging of separate genotyping batches

Supplementary Table S5. Association of classical 2-digit and 4-digit HLA alleles with JDM

Supplementary Table S6. Genome-wide imputed loci displaying potential association with JDM, with p-value cut-offs of 1×10^6^ and minor allele frequency 0.01-0.05

**List of figures:**

Supplementary Figure S1. Quantile-quantile (QQ) plot of p-values from logistic regression analysis of association of SNPs with JDM

Supplementary Figure S2. Location of amino acid position 37 within HLA-DRβ1, relative to positions implicated in other autoimmune diseases

Supplementary Figure S3. Regional association plots for rs6501160 and rs6892006.

Supplementary Figure S4. Manhattan plot of the association of imputed SNPs with JDM

Supplementary Figure S5. PCA of LD-pruned SNPs for cases and controls

Supplementary Figure S6. Allele frequency correlation plot for genome-wide imputation

**Additional supplementary data:**

HLA_classical_and_amino_acid_summary_statistics.xlsx is a separate file containing the summary statistics for the association of classical 2- and 4-digit alleles and amino acid alleles with JDM

**Supplementary Table S1. Demographic features of n=851 patients contributing samples after quality control**

| **Feature** | **Median [interquartile range] or count (percentage)** |
| --- | --- |
| Sex^a^  Female  Male | 517 (69%)  237 (31%) |
| Age at onset (years)^b^ | 6.4 [4.0-9.8] |
| Age at diagnosis (years)^c^ | 7.4 [4.6-11.0] |

^a^Complete data available for n=754 (89%); percentages calculated for cases where sex was recorded

^a^Complete data available on n=742 (87%)

**Supplementary Table S2. Numbers of markers following quality control steps**

|  | **JDM Batch 1** | | **JDM Batch 2** | | **JDM Batch 3** | | **Control** | |
| --- | --- | --- | --- | --- | --- | --- | --- | --- |
|  | **Removed** | **Remaining** | **Removed** | **Remaining** | **Removed** | **Remaining** | **Removed** | **Remaining** |
| Before quality control of SNPs | - | 547,644 | - | 551,839 | - | 551,004 | - | 521,950 |
| Y chromosome and mitochondrial SNPs | 2,277 | 545,367 | 2,367 | 549,472 | 2,487 | 548517 | 1,270 | 520,680 |
| < 98% call rate | 7,227 | - | 19,365 | - | 79,842 | - | 47,420 | - |
| HWE (p < 0.0001) | 6,568 | - | 1,186 | - | 4,844 | - | 345 | - |
| MAF < 0.01 | 241,159 | - | 257,512 | - | 243,691 | - | 251,374 | - |
| After quality control of SNPs | - | 293,019 | - | 277869 | - | 236,631 | - | 240,704 |
| Alignment to HRC reference genome | 8,924 | 284,095 | 39,958 | 237,911 | 9,224 | 227,407 |  | 239,408 |
| Merging of overlapping SNPs | - | 178,164 | - | 178,164 | - | 178,164 | - | 178,164 |

**Supplementary Table S3. Numbers of samples following quality control steps performed on separate genotyping batches**

|  | **JDM Batch 1** | | **JDM Batch 2** | | **JDM Batch 3** | | **Control** | |
| --- | --- | --- | --- | --- | --- | --- | --- | --- |
|  | **Removed** | **Remaining** | **Removed** | **Remaining** | **Removed** | **Remaining** | **Removed** | **Remaining** |
| Before quality control of samples | - | 446 | - | 288 | - | 359 | - | 12,474 |
| Elevated missing data (> 5%) | 5 | 441 | 5 | 283 | 18 | 341 | 4 | 12,470 |
| Outlying heterozygosity rate (> 5 sd +/- mean) | 1 | 440 | 0 | 283 | 0 | 341 | 60 | 12,410 |

**Supplementary Table S4. Numbers of samples following quality control steps performed after merging of separate genotyping batches**

|  | **JDM Batch 1** | | **Control** | |
| --- | --- | --- | --- | --- |
|  | **Removed** | **Remaining** | **Removed** | **Remaining** |
| Before quality control of samples | - | 1064 | - | 12,474 |
| Ethnic outliers | 142 | 922 | 242 | 12,232 |
| Duplicated or related individuals | 71 | 851 | 0 | 12,232 |


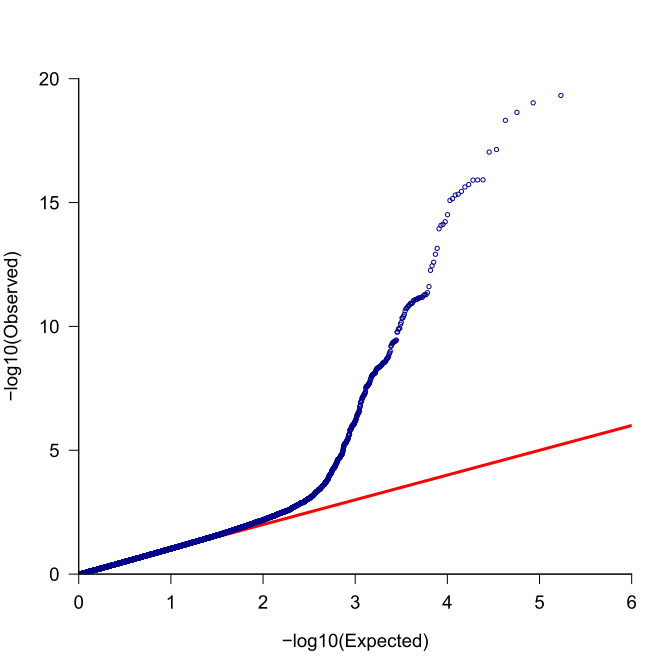


**Supplementary Figure S1. Quantile-quantile (QQ) plot of p-values from logistic regression analysis of association of SNPs with JDM.** The first 10 principal components representing population stratification were adjusted for during analysis.

**Supplementary Table S5. Association of classical 2-digit and 4-digit HLA alleles with JDM**

| **Allele** | **Odds Ratio** | **95% Confidence Interval** | **P-value** |
| --- | --- | --- | --- |
| *HLA-DQB1*02:01* | 1.79 | 1.56, 2.05 | 5.1×10^-17^ |
| *HLA-DRB1*03* | 1.66 | 1.46, 1.89 | 1.4×10^-14^ |
| *HLA-DRB1*03:01* | 1.66 | 1.46, 1.89 | 1.4×10^-14^ |
| *HLA-DQA1*05* | 1.41 | 1.27, 1.56 | 2.7×10^-10^ |
| *HLA-DQA1*05:01* | 1.41 | 1.27, 1.56 | 2.7×10^-10^ |
| *HLA-C*0701* | 1.47 | 1.30, 1.66 | 6.4×10^-10^ |
| *HLA-C*02* | 1.79 | 1.47, 2.19 | 1.0×10^-8^ |
| *HLA-C*02:02* | 1.79 | 1.47, 2.19 | 1.0×10^-8^ |
| *HLA-DQA1*01* | 0.75 | 0.67, 0.8 | 3.6×10^-8^ |
| *HLA-DRB1*15* | 0.66 | 0.57, 0.78 | 6.5×10^-7^ |
| *HLA-DRB1*15:01* | 0.66 | 0.57, 0.78 | 6.5×10^-7^ |
| *HLA-DRB1*07* | 0.67 | 0.57, 0.79 | 2.8×10^-6^ |
| *HLA-DRB1*07:01* | 0.67 | 0.57, 0.79 | 2.8×10^-6^ |
| *HLA-DQB1*02* | 1.32 | 1.18, 1.49 | 3.5×10^-6^ |
| *HLA-DQB1*06:02* | 0.74 | 0.65, 0.84 | 4.7×10^-6^ |

Significant alleles are presented here. Summary statistics for all classical 2-digit and 4-digit HLA alleles and amino acid alleles can be found in supplementary file: HLA_classical_and_amino_acid_summary_statistics.xlsx

**
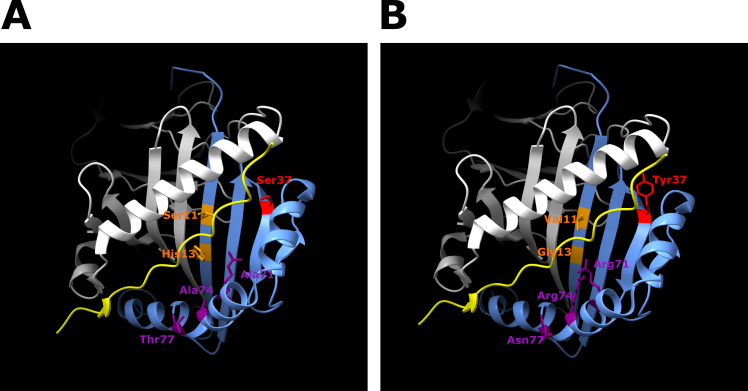
**

**Supplementary Figure S2. Location of amino acid position 37 within HLA-DRβ1, relative to positions implicated in other autoimmune diseases. (A)** Residue Ser37 which is protective for JDM is highlighted in red, with protective residues for other autoimmune diseases highlighted as indicated: Ser11 (orange), His13 (orange), Ala71 (purple), Ala74 (purple) and Thr77 (purple). **(B)** Residue Tyr37 which confers risk for JDM is highlighted in red, with risk residues for other autoimmune diseases highlighted as indicated: Val11 (orange), Gly13 (orange), Arg71 (purple), Arg74 (purple) and Asn77 (purple). The β-chain of the HLA-DR complex is coloured blue, with the α- and γ-chains coloured white and yellow, respectively. Molecular structure of HLA-DR visualised using ChimeraX 1.0.

**Supplementary Table S6. Genome-wide imputed loci displaying potential association with JDM, with p-value cut-offs of 1×10^6^ and minor allele frequency 0.01-0.05^a^**

| **Rsid^b^** | **Chr** | **Position** | **Allele A** | **Allele B** | **MAF** | **OR** | **95% CI** | **p-value** | **Imputation R^2 c^** | **Info measure^d^** | **Association info measure^e^** | **Nearest assayed rsid^f^** | **R^2^ nearest assayed rsid** | **p-value nearest assayed rsid** |
| --- | --- | --- | --- | --- | --- | --- | --- | --- | --- | --- | --- | --- | --- | --- |
| rs139672953 | 1 | 90779678 | T | G | 0.01 | 5.10 | 2.98,8.71 | 2.6×10^-9^ | 0.82 | 0.82 | 0.68 | rs4446947 | 0.009 | 0.47 |
| rs140108689 | 2 | 105595719 | T | C | 0.01 | 4.23 | 2.59,6.89 | 7.8×10^-9^ | 0.94 | 0.94 | 0.93 | rs7608291 | 0.01 | 0.84 |
| rs73958160 | 2 | 96748169 | G | C | 0.02 | 3.39 | 2.23,5.15 | 1.1×10^-8^ | 0.71 | 0.71 | 0.68 | - | - | - |
| rs116386365 | 6 | 52068835 | C | T | 0.01 | 6.28 | 3.32,11.9 | 1.7×10^-8^ | 0.62 | 0.62 | 0.41 | rs2397079 | 0.002 | 0.15 |
| rs78829749 | 6 | 6594485 | C | T | 0.01 | 4.48 | 2.65,7.57 | 2.3×10^-8^ | 0.85 | 0.85 | 0.79 | rs4246076 | 0.004 | 0.54 |
|  |  |  |  |  |  |  |  |  |  |  |  | rs9328377 | 0.002 | 0.39 |
| rs112016802 | 8 | 110654125 | C | T | 0.01 | 3.58 | 2.25,5.69 | 6.6×10^-8^ | 0.85 | 0.85 | 0.85 | rs11998387 | 0.02 | 0.03 |
| rs192074881 | 9 | 98054640 | G | A | 0.02 | 3.26 | 2.09,5.09 | 2.0×10^-7^ | 0.81 | 0.81 | 0.74 | - | - | - |
| rs11568763 | 9 | 101893073 | G | A | 0.02 | 3.19 | 2.04,4.97 | 3.4×10^-7^ | 0.67 | 0.67 | 0.61 | - | - | - |
| rs182199585 | 19 | 49064197 | C | G | 0.03 | 2.59 | 1.78,3.76 | 5.7×10^-7^ | 0.65 | 0.65 | 0.58 | rs12611137 | 0.05 | 0.13 |
| rs7255994 | 19 | 48827110 | T | C | 0.04 | 1.94 | 1.49,2.53 | 9.4×10^-7^ | 0.98 | 0.98 | 0.97 | rs8182501 | 0.03 | 0.64 |

^a^Loci with p-values below a suggestive level of significance (1×10^6^) are displayed for alleles with minor allele frequency (MAF) between 0.01 and 0.05

^b^Rsid, Reference SNP cluster ID

^c^Squared Correlation of imputation of genotypes with true unmeasured genotypes, as estimated by Minimac3 during imputation via the Michigan Imputation Server: https://genome.sph.umich.edu/wiki/Minimac3_Info_File#Rsq

^d^Impute INFO measure calculated by SNPTEST: https://mathgen.stats.ox.ac.uk/genetics_software/snptest/snptest.html#info_measures

^e^Relative information measure about parameters of the model fitted during association testing: https://mathgen.stats.ox.ac.uk/genetics_software/snptest/snptest.v2.pdf

^f^Nearest assayed marker within 100,000 kb


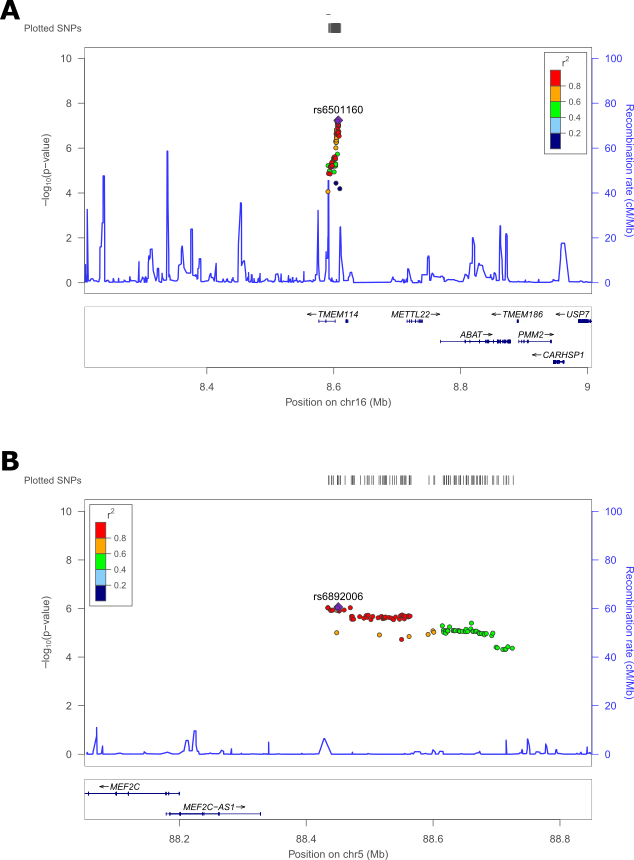


**Supplementary Figure S3. Regional association plots for (a) rs6501160 and (b) rs6892006.**

**
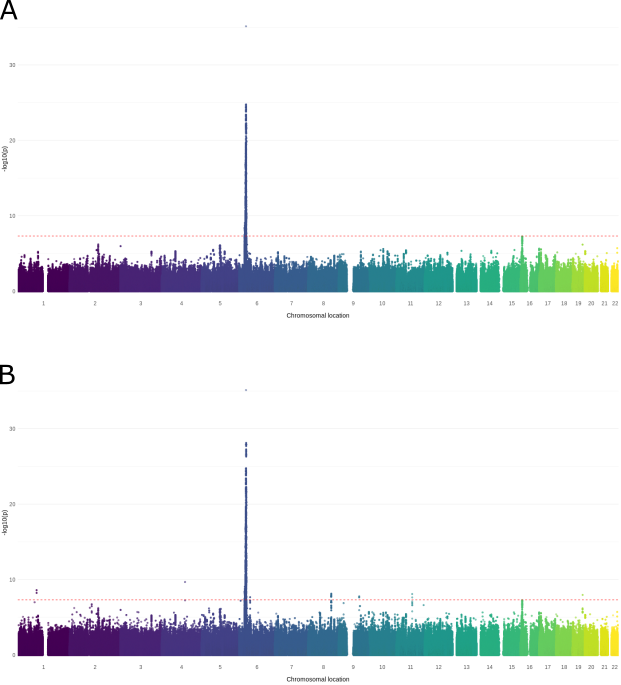
**

**Supplementary Figure S4. Manhattan plot of the association of imputed SNPs with JDM.** Loci are filtered according to minor allele frequency of 0.01. The red dotted line indicates genome-wide level of significance (5×10^-8^). The degree of transparency of each data-point represents the R^2^ value for imputation accuracy, with more solid colours representing higher certainty.


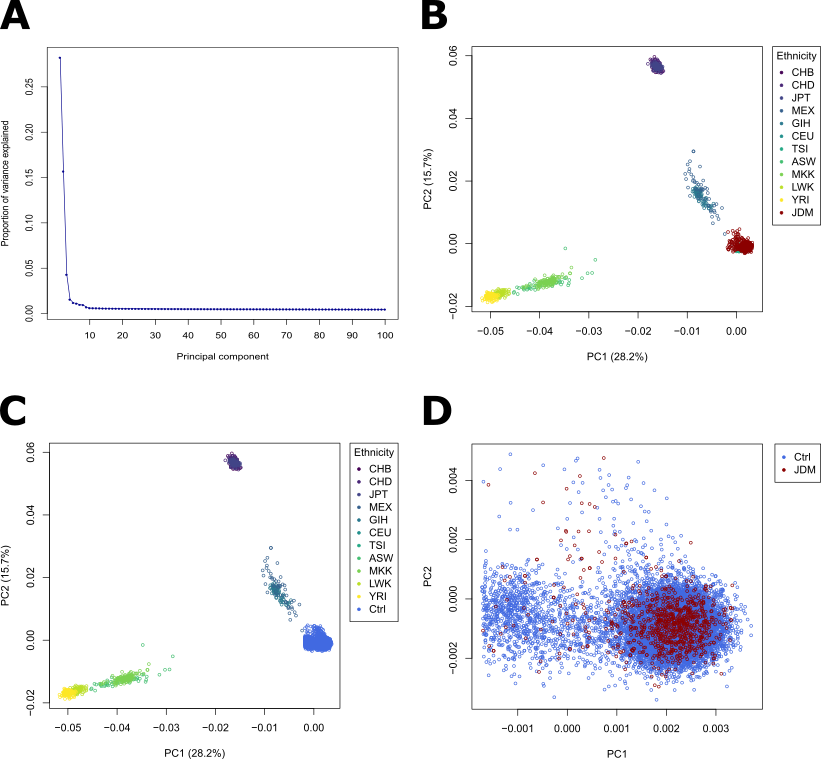


**Supplementary Figure S5. PCA of LD-pruned SNPs for cases and controls.** PCA performed on assayed JDM dataset merged with control dataset and data from the International HapMap 3 representing diverse ethnic populations. **(A)** Proportion of variance explained by 1-100 PCs. **(B)** Selected JDM cases (red) with the HapMap populations plotted for the first 2 PCs. **(C)** Selected controls (blue) with the HapMap populations plotted for the first 2 PCs. **(D)** Matching of cases and controls. The genomic inflation factor (λ_GC_) was calculated as 1.09 using PLINK 1.07. λ_1000_ was calculated as 1.00 using the formula *λ*= 1 + 500 × (*λ* - 1) × (1/N+ 1/N) [41].


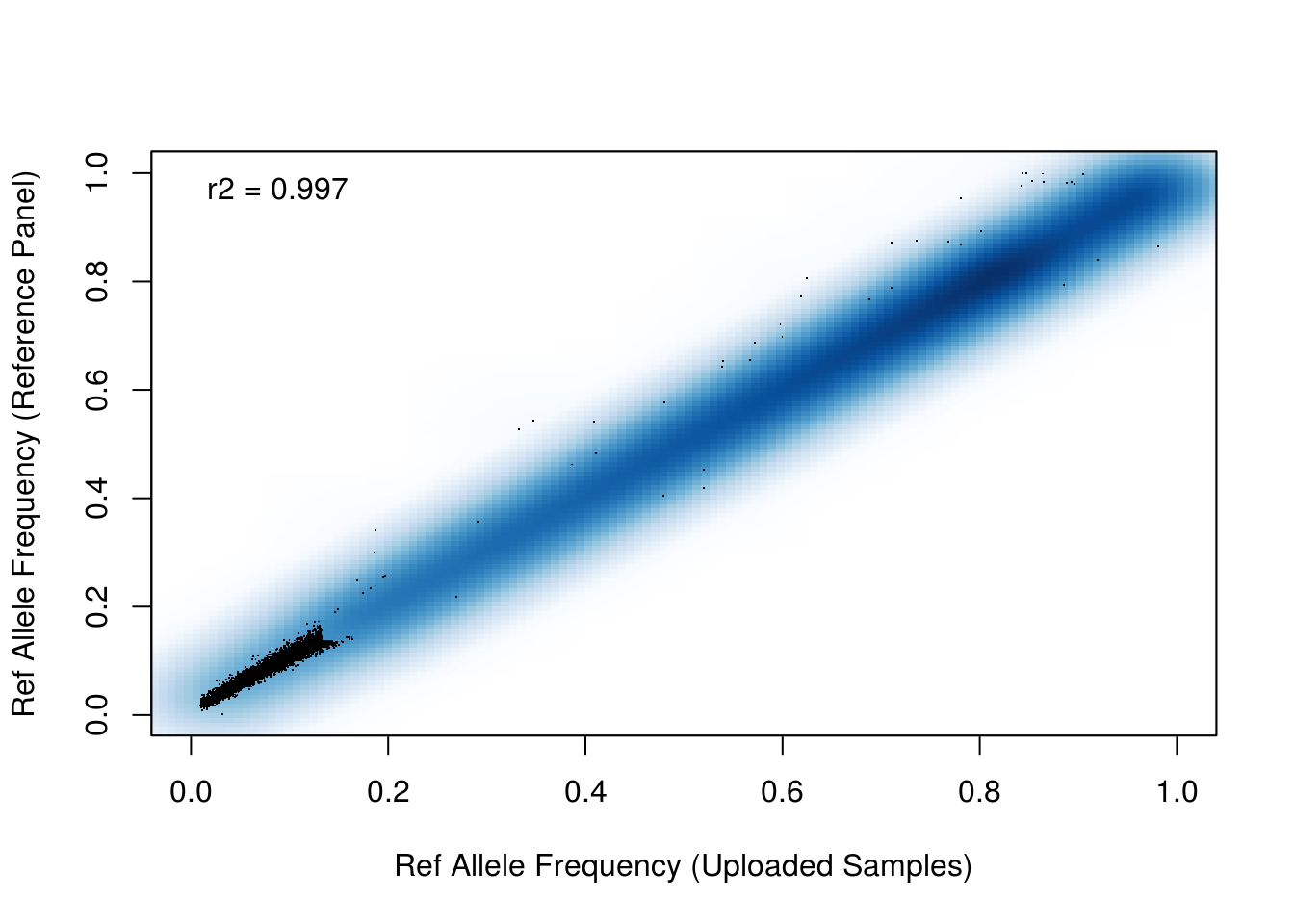


**Supplementary Figure S6. Allele frequency correlation plot for genome-wide imputation.** Allele frequencies for uploaded samples visualised against allele frequencies for the reference panel for the first 5000 points from areas of lowest regional densities. A total of 113 mismatches where chi-squared was greater than 300 were identified.
